# Modified Methacrylate Hydrogels Improve Tissue Repair after Spinal Cord Injury

**DOI:** 10.3390/ijms19092481

**Published:** 2018-08-22

**Authors:** Aleš Hejčl, Jiří Růžička, Kristýna Kekulová, Barbora Svobodová, Vladimír Proks, Hana Macková, Kateřina Jiránková, Kristýna Kárová, Lucia Machová Urdziková, Šárka Kubinová, Jiří Cihlář, Daniel Horák, Pavla Jendelová

**Affiliations:** 1 Institute of Experimental Medicine, Academy of Sciences of the Czech Republic, Vídeňská 1083, 142 20 Prague, Czech Republic; stoupa-ruza@seznam.cz (J.R.); kristyna.kekulova@iem.cas.cz (K.K.); barbora.svobodova@iem.cas.cz (B.S.); kristyna.karova@iem.cas.cz (K.K.); urdzikl@saske.sk (L.M.U.); sarka.kubinova@iem.cas.cz (S.K.); pavla.jendelova@iem.cas.cz (P.J.); 2 Department of Neurosurgery, J. E. Purkinje University, Masaryk Hospital, Sociální Péče 12A, 401 13 Ústí nad Labem, Czech Republic; 3 Institute of Macromolecular Chemistry, Academy of Sciences of the Czech Republic, Heyrovského nám.2, 162 06 Praha, Czech Republic; proks@imc.cas.cz (V.P.); mackova@imc.cas.cz (H.M.); horak@imc.cas.cz (D.H.); 4 Second Faculty of Medicine, Charles University, V Úvalu 84, 150 06 Prague, Czech Republic; jirankova.katerina@seznam.cz; 5 Department of Neuroscience, 2nd Faculty of Medicine, Charles University, V Úvalu 84, 150 06 Prague, Czech Republic; 6 Department of Mathematics, Faculty of Science, J. E. Purkyně University, České Mládeže 8, 400 96 Ústí nad Labem, Czech Republic; jiri.cihlar@ujep.cz

**Keywords:** spinal cord injury, hydrogel, connective tissue, neurofilaments, locomotor test, plantar test

## Abstract

Methacrylate hydrogels have been extensively used as bridging scaffolds in experimental spinal cord injury (SCI) research. As synthetic materials, they can be modified, which leads to improved bridging of the lesion. Fibronectin, a glycoprotein of the extracellular matrix produced by reactive astrocytes after SCI, is known to promote cell adhesion. We implanted 3 methacrylate hydrogels: a scaffold based on hydroxypropylmethacrylamid (HPMA), 2-hydroxyethylmethacrylate (HEMA) and a HEMA hydrogel with an attached fibronectin (HEMA-Fn) in an experimental model of acute SCI in rats. The animals underwent functional evaluation once a week and the spinal cords were histologically assessed 3 months after hydrogel implantation. We found that both the HPMA and the HEMA-Fn hydrogel scaffolds lead to partial sensory improvement compared to control animals and animals treated with plain HEMA scaffold. The HPMA scaffold showed an increased connective tissue infiltration compared to plain HEMA hydrogels. There was a tendency towards connective tissue infiltration and higher blood vessel ingrowth in the HEMA-Fn scaffold. HPMA hydrogels showed a significantly increased axonal ingrowth compared to HEMA-Fn and plain HEMA; while there were some neurofilaments in the peripheral as well as the central region of the HEMA-Fn scaffold, no neurofilaments were found in plain HEMA hydrogels. In conclusion, HPMA hydrogel as well as the HEMA-Fn scaffold showed better bridging qualities compared to the plain HEMA hydrogel, which resulted in very limited partial sensory improvement.

## 1. Introduction

Functional deficits of spinal cord injury (SCI) are the result of subsequent temporal events: the primary insult leads to ischemia, followed by neuronal cell death, axon damage, and demyelination. Subsequently, glial activation, the release of inflammatory factors and cytokines, and the scar formation that prevents axons to regenerate leads to progression of the lesion. Post-traumatic syringomyelia usually develops in the chronic phase after SCI as a result of hemorrhage and tissue necrosis. The cavity is filled with tissue debris and later mostly with CSF and is surrounded by glial and mesenchymal scarring forming a barrier for tissue regeneration. Therefore, different strategies have been developed to treat SCI. One of the approaches relies on tissue engineering methods, particularly on bridging the lesion cavities. The scaffold, suitable for implantation into lesion cavity must have the appropriate chemical, physical, and mechanical properties required for cell survival and tissue formation. One of the most suitable classes of compounds for these purposes is definitely represented by hydrogels [1,2,3]. They are three-dimensional (3D) hydrophilic polymers held together by covalent bonds or other cohesive forces such as hydrogen or ionic bonds [3,4,5]. They can be either synthetic or natural in origin, or a combination of both. Synthetic polymers can be tailored in terms of composition, rate of degradation, and mechanical and chemical properties. [6]. This is much more difficult to achieve in naturally derived polymers, which, in contrast, have features supporting adhesion and cell growth.

Therefore, the presence of bioadhesive and bioactive molecules on an artificial hydrogel matrix is crucial for the successful preparation of neural biomimetic scaffolds. Synthetic materials are often coated or modified with extracellular matrix (ECM) components, e.g., laminin and fibronectin, or synthetic peptides [7,8,9], which can improve cell adhesion and survival by generating a permissive microenvironment within the biomaterial [10,11]. Moreover, collagen, fibronectin, and laminin are associated with wound healing and regeneration and, therefore, they can be explored for therapeutic purposes. After SCI, fibronectin is deposited within the glial scar [12]. Yet fibronectin is also known to play a role in cell differentiation, proliferation, or migration and has been used as an implant in a spinal cord lesion in some studies [13]. Fibronectin is an important glycoprotein in the developing CNS due to its involvement in cell migration [14] and it has important roles in tissue repair mainly because of its cell adhesion properties. Fibronectin was used as matrix for NSC transplants in damaged CNS and found to improve NSC survival [15]. Fibronectin can be also used in combination with other materials. For instance, poly-β-hydroxybutyrate fibers coated with alginate hydrogel and fibronectin were used for a Schwann cell transplant in the injured rat spinal cord [13]. This combination promoted axon growth across the injury.

During the last 15 years we have implanted various biomaterials inside cavities in order to provide a scaffold for the ingrowth of new tissue, especially axons and blood vessels [16,17,18,19,20,21]. In general, we have shown that hydrogels are able to provide scaffolding for new tissue to grow inside their pores. As with other studies, we have also shown that the quantity of tissue ingrowth is influenced by various adjustments of tissue scaffold, such as in combination with adhesion molecules, modifications in chemical and physical properties, or in combination with stem cells [22,23,24,25]. Such modifications may lead to motor and sensory function improvement in experimental rats after SCI [25]. Despite some improvements, the overall results are unsatisfactory, impelling the development of new scaffolds with better proregenerating qualities. In this study we implanted methacrylate hydrogel based on poly(2-hydroxyethyl methacrylate) (HEMA) modified with attached fibronectin (HEMA-Fn) and compared it with unmodified HEMA and poly[*N*-(2-hydroxypropyl) methacrylamide] (HPMA) hydrogel scaffolds in terms of functional response and tissue infiltration. We utilized a spinal cord hemisection, which provides an appropriate model for evaluating scaffolds with oriented pores as the lesion is large enough for proper orientation of the scaffold within the cavity and at the same time the functional deficit is mild enough so as to avoid excessive animal loss as opposed to other more severe experimental lesions, such as spinal cord transection or balloon-induced compression lesion. In this study we wanted to assess the long-term effect of fibronectin-enriched HEMA and compare it with plain HEMA and HPMA hydrogels. Hydrogels with oriented pores were utilized as they provide an excellent scaffold for the evaluation of newly growing axons and blood vessels within the biomaterial.

## 2. Results

### 2.1. Hydrogel Scaffold

**Figure 1:**
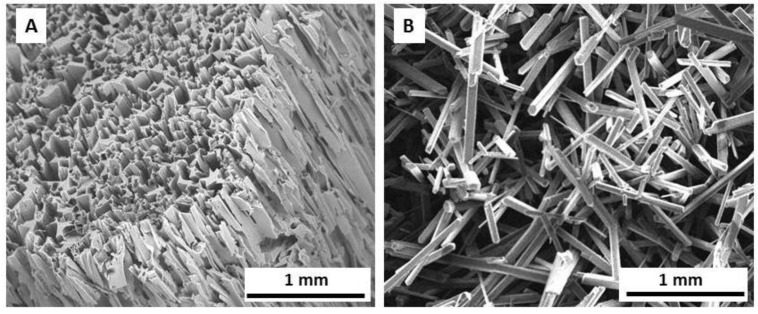
SEM micrographs of HEMA hydrogel (**A**) and ammonium oxalate crystals (**B**).

Three synthetic hydrogels, namely HEMA, HEMA-Fn, and HPMA, were obtained by free-radical copolymerization producing identical inner morphology, exemplified in Figure 1A. Longitudinally shaped pores were imprinted by needle-like ammonium oxalate crystals oriented in an axial direction (Figure 1B). The pore size corresponded to the size of the starting crystals, which were 30–90 µm thick and approximately 0.3–10 mm long. According to published literature [26], this long pore size can be advantageous for the regeneration of peripheral axons, whereas their ingrowth and/or outgrowth needs much smaller pores (20–70 μm) depending on the origin of biomaterial*.* The pore volume calculated from an oxalate/monomer volume ratio, amounted to ~70%, which roughly corresponded to Hg porosimetry data (68.4%) [22]. The elasticity modulus of the hydrogels was ~4 and 30 kPa perpendicularly and along the pores, respectively [27].

### 2.2. Functional Tests

All 34 animals underwent functional evaluation. The animals were tested for motor function and sensory function of the hindlimbs. The motor function was tested using the BBB score and sensory function using the plantar test. Considering the BBB testing, the right hindlimb was the injured one while the left hindlimb was considered the uninjured one. However, for the plantar test the left hindlimb was considered the injured one due to crossing of the spino-thalamic tract and the right hindlimb was considered uninjured.

### 2.3. BBB

**Figure 2:**
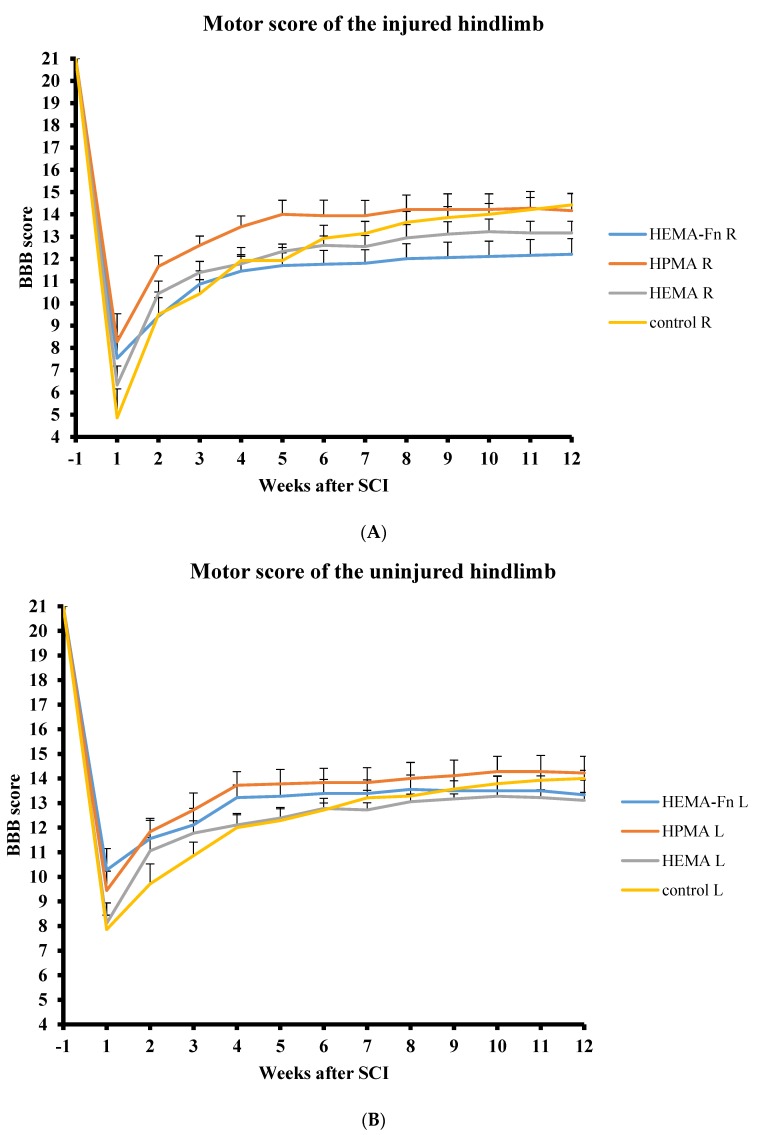
Comparison of motor function evaluation of the injured (**A**) and uninjured (**B**) side of hindlimbs using the BBB score. There were no statistically significant differences among the four groups (three treatment groups and one control group-hemisection only). Note the lower scores of the injured hindlimb on the side of the hemisection. The data represent mean values and error bars represent SEM.

All the animals in the study were pre-evaluated before surgery and then tested every week until week 12 after hydrogel implantation. The BBB scores for both legs were compared between the control group and the three groups of animals treated with hydrogels. During the 12 week period there were no statistically significant differences in the BBB scores for the left and right hindlimbs among any of the 4 groups (Figure 2).

### 2.4. Plantar Test

**Figure 3:**
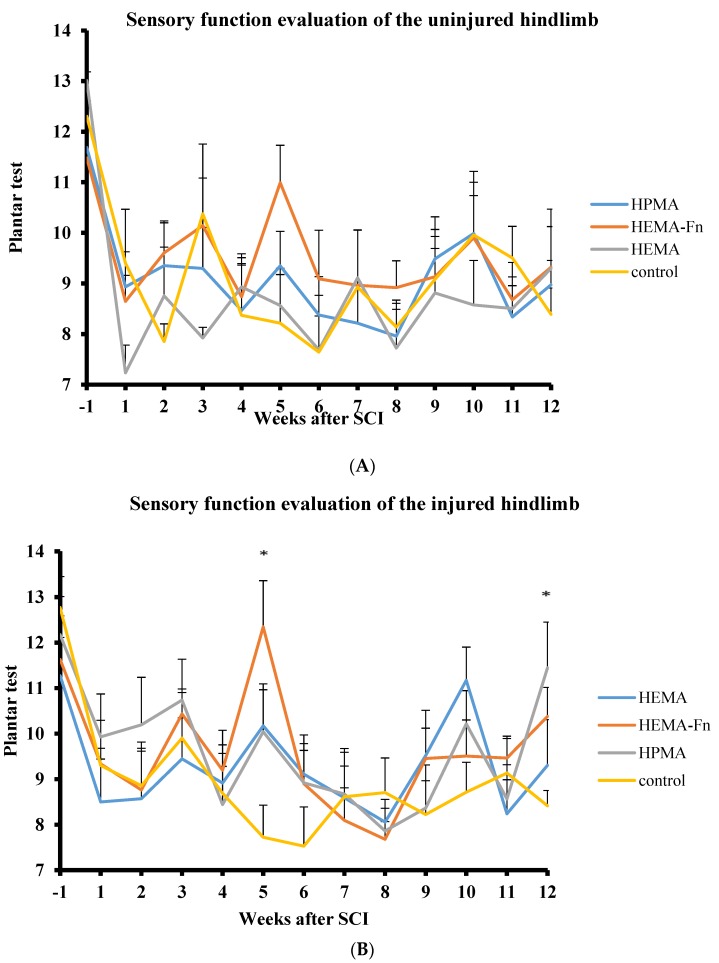
Sensory function evaluation of the uninjured (**A**) and injured (**B**) side of hindlimbs using the plantar test. There were statistically significant differences in the sensory response between rats treated with 2-hydroxyethylmethacrylate (HEMA)-Fn and the control group on week 5 and then between the HPMA and both the HEMA and the control group on week 12 (* *p* < 0.05). The data represent mean values and error bars represent SEM.

The animals were pretreated twice before surgery and then evaluated once a week until week 12 after hydrogel implantation. The plantar test for the right leg (uninjured side, Figure 3A) was not statistically significant for any of the time period. However, for the left side (injured one, Figure 3B), we found a statistically significant difference between the HEMA-Fn and the hemisection group in week 5. Furthermore, there was a statistically significant difference between the HPMA hydrogel-treated group and the hemisection group and the HPMA-treated group and the HEMA-group in week 12.

### 2.5. Microscopic Evaluation of the Hydrogel Bridge

**Figure 4:**
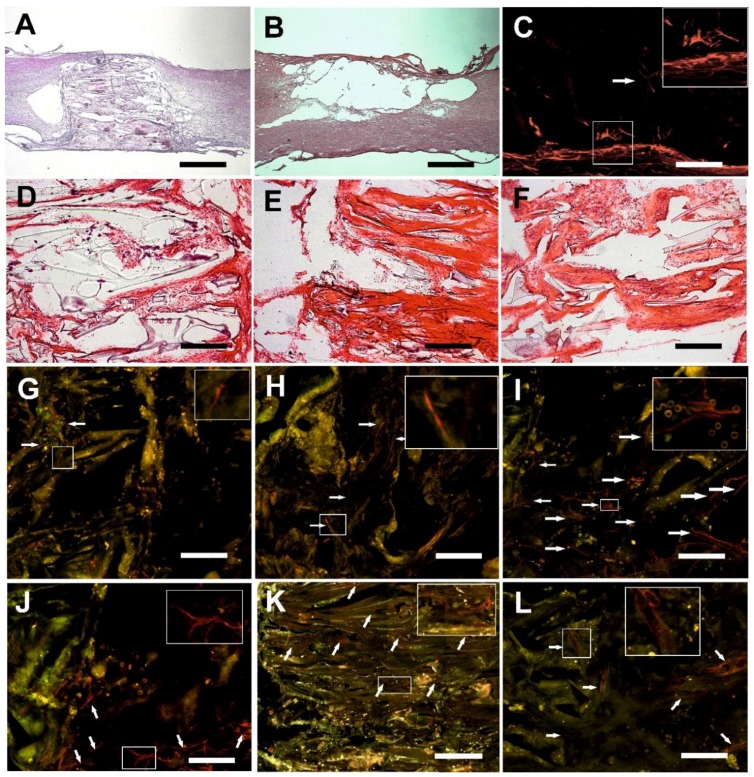
The mosaic presents various aspect of tissue ingrowth within the pores of the three methacrylate hydrogels. (**A**) HEMA-Fn sufficiently bridged the hemisection cavity. In some cases there were minor cavities on the border. The HEMA-Fn scaffold serves as a representative sample for all three methacrylate hydrogels (HE staining, scale bar = 1 mm). (**B**) The lesion resulted in a large pseudocystic cavity in the control group (HE staining, scale bar = 1 mm). (**C**) Subtle astrogliosis was present within the spinal cord tissue neighboring the HPMA scaffold and a few islets of astrocytes also infiltrated its peripheral part (white arrow, GFAP-Cy3 staining, scale bar = 100 µm). (**D**) Sparse connective tissue elements were found in the plain HEMA scaffold; there was a more dense infiltration within the pores of the HEMA-Fn (**E**) and the HPMA hydrogel (**F**) HE staining, scale bar = 100 µm. (**G**) No axonal sprouts were present in the HEMA scaffold, however, some axons (white arrows) reached the border zone of the spinal cord (NF160-g594 staining, scale bar = 25 µm). (**H**) A small number of sprouts (white arrows) were present within the HEMA-Fn scaffold (NF160-g594 staining, scale bar = 25 µm); significantly higher number of sprouts was present (white arrows) within the central parts (**I**) as well as the periphery (**J**) of the HPMA hydrogels (NF160-g594 staining, scale bar = 25 µm). (**K**) Blood vessels (white arrows) grew abundantly within the pores of all three hydrogels especially the HEMA-Fn scaffold, as seen in this image (RECA-g488 staining, scale bar = 50 µm). (**L**) A detailed view of the blood vessels within the HEMA-Fn scaffold (RECA-g488 staining, scale bar = 25 µm). Insert boxes depict the typical elements described in each image of the mosaic (enlarged twice to the original size).

All three hydrogel scaffolds bridged the hemisection lesion providing scaffold for tissue regrowth. In some cases small residual cysts were present on the border between the hydrogel and the spinal cord (Figure 4A). There were no signs of foreign body reactions observed in or around the hydrogels 3 months after implantation. In the control group, the lesion was represented by a large cavity with no tissue inside (Figure 4B). There was a slight narrow rim of astrogliosis with small isles of astrocytes entering the border zones of all three hydrogel scaffolds (Figure 4C). We did not observe any difference despite the fact that plain HEMA resulted in upregulation of GFAP gene (see section Gene Expression). No signs of foreign body-type giant-cell granulomatous reaction were observed 3 months after hydrogel implantation. We found no statistically significant difference in the markers of immune response among any of the 3 scaffolds (see section Gene Expression).

### 2.6. Connective Tissue

**Figure 5:**
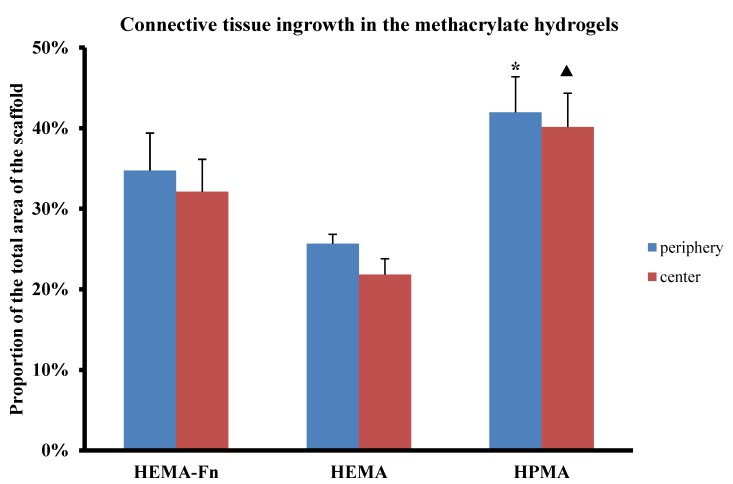
Comparison of connective tissue ingrowth within the three hydrogels. The graph presents the percentage of HE-positively stained tissue within the whole area of the peripheral (blue bars) and central (red bars) regions of the hydrogel. The HPMA scaffold showed higher connective tissue ingrowth within the periphery (* *p* < 0.05) as well as the central part (▲ *p* < 0.05) of the HEMA scaffold. Data shown as mean, and the error bars represent SEM. For statistical analysis, a one-way ANOVA test was used.

All 3 types of hydrogels were filled with connective tissue, however there were obvious differences. Only a little dispersed connective tissue was present in the pores of the plain HEMA hydrogels (Figure 4D). Alternatively, in both the HPMA and the HEMA-Fn hydrogels, connective tissue elements were more abundant with a rather dense infiltration of the pores (Figure 4E,F). The HPMA hydrogel promoted connective tissue infiltration inside the scaffold, in both its peripheral as well as the central part, compared to the plain HEMA scaffold (Figure 5, *p* < 0.05). The HEMA scaffold with attached fibronectin showed tendency towards higher connective tissue infiltration in both the peripheral and the central part of the hydrogel without reaching statistical significance (Figure 5, *p* < 0.05).

### 2.7. Axonal Regeneration

**Figure 6:**
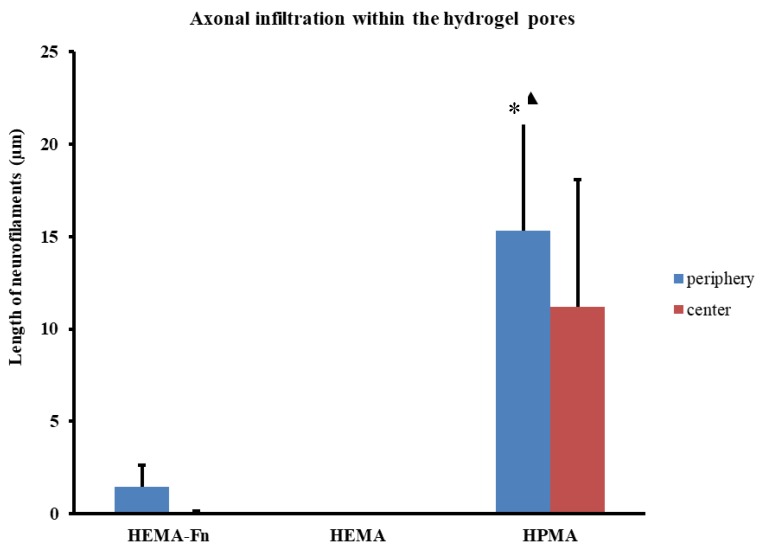
Comparison of axonal ingrowth within the three hydrogels. The total length of axons was assessed within the peripheral (blue bars) as well as the central (red bars) regions of the scaffold. The graph shows that the HPMA scaffold promoted statistically significant infiltration of axons within the pores of the scaffold compared to both HEMA hydrogels (* *p* < 0.05, ▲ *p* < 0.05). The graph shows that there were no axons within the HEMA scaffold, while the modification of fibronectin (HEMA-Fn) showed at least some axonal infiltration with the periphery and some even grew as far as the central parts of the scaffold. Data shown as mean and the error bars represent SEM. For statistical analysis, a one-way ANOVA test was used.

After 3 months, HPMA hydrogels were infiltrated with axonal sprouts in the peripheral areas as well as in the central parts (Figure 6). The growth of axons was guided by the oriented pores, mostly in the cranio-caudal and caudo-cranial direction. The plain HEMA scaffold did not show any neurofilaments in its pores 3 months after implantation (Figure 4G), despite its upregulation of Gap43 (see section Gene Expression). In contrast, the HEMA-Fn hydrogels showed that some axons grew into the peripheral parts of the scaffold and a minimal amount also reached the central parts of the scaffold (Figure 4H). The amount of neurofilaments in the HPMA hydrogel was significantly higher compared to both HEMA-based scaffolds (Figure 4I). There were many new axons in the peripheral part of the HPMA scaffold which extended all the way to its central area (Figure 4J).

### 2.8. Growth of Blood Vessels in the Hydrogels

**Figure 7:**
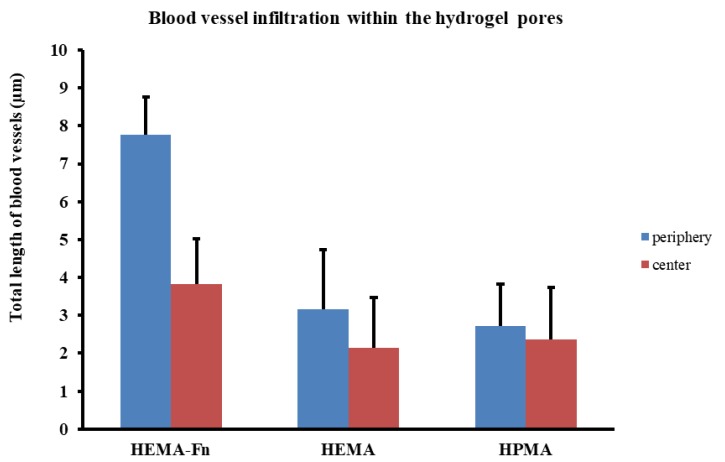
Comparison of blood vessels in growth within the three hydrogels. The total length of blood vessels was assessed within the peripheral (blue bars) as well as the central (red bars) regions of the scaffold. There was no statistically significant difference between the three groups but the HEMA-Fn scaffold showed a trend towards increased ingrowth of blood vessels within its pores, especially in the peripheral areas. Data shown as mean and the error bars represent SEM. For statistical analysis, a one-way ANOVA-test was used.

All three hydrogels showed an extensive ingrowth of blood vessels into the periphery as well as the central part of the scaffolds—both the HEMA-based hydrogels as well as the HPMA (Figure 4K,L). Despite not reaching statistical significance, the HEMA-based hydrogel modified with fibronectin supported the highest number of blood vessels compared to the plain HEMA and the HPMA scaffolds (Figure 7). There was also no difference in the VEGFA gene during molecular analysis (see section Gene Expression). As with the axons, blood vessels also grew predominantly in an oriented fashion guided by the pores

### 2.9. Gene Expression

**Figure 8:**
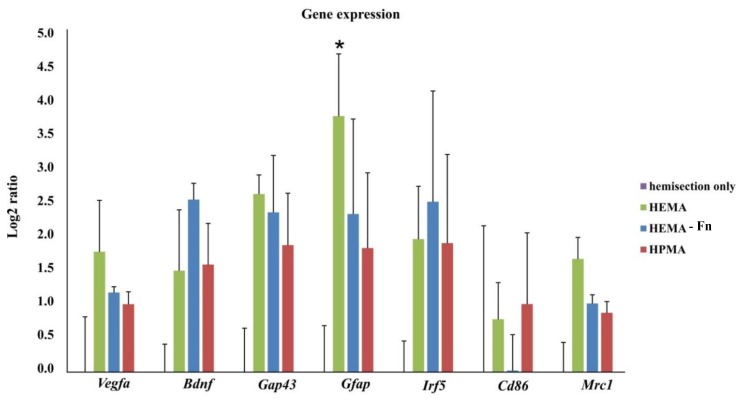
Gene expression in spinal cord tissue after treatment of hemisection with 3 different methacrylate scaffolds. Treatment with HEMA resulted in significant upregulation of Gfap when compared with controls (hemisection only, *n* = 3). We found no other statistically significant difference in any markers within the tissue of spinal cords treated with the methacrylate hydrogels or controls. Data shown as mean and SEM relative to hemisection (injured tissue), which was set as 0 (with * *p* < 0.05 (vs. hemisection)). For statistical analysis a one-way ANOVA test was used.

Expression of genes related to regeneration (Vegfa, Bdnf, Gap43), glial scaring (Gfap) and infiltrating macrophage phenotype (Irf5, Cd86, Mrc1) was determined 3 months after hemisection or implantation of methacrylate scaffolds. Significant upregulation of Gfap genes was detected after treatment with HEMA when compared with controls. Implantation of HEMA resulted in a trend of upregulation of M2 associated Mrc1 when compared to both HEMA-Fn and HPMA. No other statistically significant difference in any markers within the tissue of spinal cords treated with the methacrylate hydrogels or controls were observed (Figure 8).

## 3. Discussion

SCI, especially at its chronic stage, is characterized by glial scarring and pseudocystic cavities associated with disruption of long spinal cord tracts. Experimental studies using a variety of biomaterials, including hydrogels, have been conducted during the last 20 years [16]. In this study we evaluated two methacrylate hydrogels based on HEMA and HPMA, which are considered to be excellent synthetic biomaterials resembling living tissue, in terms of water content and mechanical properties [28,29]. Their porous properties, introduced by polymerization of the respective monomers in the presence of inorganic needle-like crystals, make them suitable for neural tissue or spinal cord reconstruction as has been shown in many studies [16,20,30,31]. The advantage of synthetic hydrogels consists not only of their biological inertness, but also in the variety of possible modifications, allowing better tissue repair promoting properties [23,32,33]. Our study showed that none of the methacrylate hydrogels caused a significant immune response, as demonstrated by no upregulation of antigen-presenting cells at 3 months. There were increased signs of tissue repair in the plain HEMA scaffold 3 months after SCI as demonstrated by an upregulation of an Mrc1 gene.

In this study, one of the HEMA scaffolds was modified with attached fibronectin. Our previous study showed better bridging qualities of the HPMA scaffold when compared to HEMA. Based on our positive results with surface modification of scaffolds, we also compared fibronectin-modified HEMA, to test whether such surface modification would balance the advantage of the HPMA scaffold. Fibronectin is known to promote cellular migration, proliferation, and differentiation [34,35]. In this study we showed better connective tissue infiltration within the HPMA scaffold compared to HEMA. There was more connective tissue within HEMA modified with fibronectin when compared to plain HEMA 3 months after SCI but without reaching statistical significance. This is an extension of the finding by other authors, which demonstrated that fibronectin enhances short-term cell adhesion [36].

Several studies have also shown that fibronectin may be neuroprotective, reducing post-traumatic apoptosis while enhancing functional outcome [37,38]. King et al. showed that fibronectin promotes the growth of axons into the implant when compared to other natural molecules such as fibrin or collagen, but with the disadvantage of having large cavities at the spinal cord lesion borderline [36]. The combination of an artificial scaffold with fibronectin thus seems to be a useful type of combined therapy that may promote the regrowth of axons while creating a positive milieu for the regrowth of new axons. The combination with a HEMA scaffold ensured good integration of the implant within the knife-cut experimental cavity while fibronectin promoted the axons to cross the hydrogel-spinal cord border and infiltrate the pores in the periphery of the implant as well as in the central area. Nonetheless, none of the three methacrylate hydrogels in our study led to motor function improvement. We did not observe any differences in BBB test, except for lower BBB score on the ipsilateral side. The results from plantar test indicate that no further hyperalgesia has developed after the hydrogel implantation. HEMA-Fn and HPMA resulted in only inconsistent sensory function difference of the left hindlimb, despite the differences in the number of axons within the three scaffolds. Spinal cord hemisection is not an ideal model for testing of functional improvement. However, it is suitable for histological evaluation and quantification of cellular ingrowth into the implant.

We did not observe any axons extending across the whole scaffold and crossing the scaffold-tissue border back and re-entering the spinal cord. A higher number of axons infiltrating the scaffold but without making connections across the bridge does not therefore necessarily result in functionally meaningful improvement.

Previous studies have also shown that fibronectin enhances axonal ingrowth after SCI but the studies were restricted to a short time evaluation period. This may not, however, reflect long-term data as we have shown in our recent paper [39]. In our study, fibronectin modification of the HEMA scaffold showed some increased but statistically insignificant difference in the ingrowth of axons into the peripheral and also central parts, while there were no axonal sprouts present in the plain HEMA 3 months after SCI. When comparing the results based on the chemical backbone of the hydrogel (HEMA vs. HPMA), the HPMA scaffold supports the regrowth of axons much better compared to HEMA, even when the latter one is enriched with fibronectin. This is in line with our previous study, in which the HPMA scaffold showed improved axonal infiltration compared to HEMA [23]. Interestingly, molecular analysis showed a tendency towards increased sprouting in all three scaffolds. However, only HPMA hydrogel and partially HEMA-Fn create an environment which prevents the axon retraction from the hydrogel.

None of the tested scaffolds supported the ingrowth of astrocytes, though the GFAP protein was upregulated in the plain HEMA scaffold when compared with the hemisection. We observed only a slight astrogliosis around the 3 types of scaffolds.

New blood vessels grew into all three hydrogels; there were many of them throughout the periphery and growing further into the central parts. We found a tendency towards a denser blood vessel network in the HEMA-Fn hydrogel. This may be explained by the presence of the RGD (arginine–glycine–aspartate) peptide sequence within the fibronectin molecule, which has been shown to enhance vascular growth [23]. The presence of blood vessels within the lesion is vital as they promote axonal repair and enhance functional improvement [40,41].

Tissue engineering is based on creating a proregenerative environment for tissue repair within the lesion. The cells and their extensions (such as axons) should be properly guided in order to make functionally meaningful connections. The advantage of hydrogels with oriented pores is their ability to navigate the growth of new tissue along the long axis of their pores. However, solid hydrogels require a surgical opening of the spine and spinal cord during the process of implantation. Also, the hydrogels we evaluated were slightly stiffer compared to the scaffolds with randomly oriented pores used in our former studies, due to the need to properly retain the oriented inner structure [21,23,25]. This may be the cause of residual minor cavities on the border between the scaffold and the spinal cord. Injectable scaffolds, on the other hand, avoid the need for surgical incision and allow minimally-invasive implantation. They are also soft enough to adhere well to the spinal cord tissue. However, oriented pores within injectable scaffolds are more difficult to achieve, and therefore we cannot ensure targeted growth within such biomaterials [42]. Study of the synthesis of injectable scaffolds with oriented pores would be of interest in the future.

## 4. Materials and Methods

### 4.1. Hydrogel Scaffolds

2-Hydroxyethyl methacrylate (HEMA; Röhm, Germany) and ethylene dimethacrylate (EDMA; Ugilor S.A., France) were purified by distillation. 1-Amino-2-propanol,1,2-diaminoethan, methacryloyl chloride, 2,2′-azobisisobutyronitrile (AIBN), 2-aminoethyl methacrylate (AEMA) hydrochloride, tris (2-carboxyethyl) phosphine hydrochloride (TCEP), and *N*-γ-maleimidobutyryloxysuccinimide ester (GMBS) were purchased from Sigma-Aldrich (St. Louis, USA). Ammonium oxalate (Lach-Ner; Neratovice, Czech Republic) was crystallized from water until the formation of 30–90 µm thick and approximately 0.3–10 mm long needle-like crystals, which were used as a porogen. Fibronectin (Fn) from human plasma was obtained from Roche (Mannheim, Germany). All other chemicals were from Lach-Ner. Ultrapure Q water ultrafiltered on a Milli-Q Gradient A10 system (Millipore; Molsheim, France) was used for preparation of phosphate buffers saline (PBS) and all other experiments. *N*-(2-hydroxypropyl)methacrylamide (HPMA) and *N*,*N*′-ethylenebis(acrylamide) (EDMAAm) were synthetized by modification of published procedure [26]. Briefly, 1-amino-2-propanol (22.5 g), NaNO_2_ (200 mg), and NaOH 12 g were dissolved in H_2_O (300 mL), cooled at 0 °C, and dry ice (40 g) and methacryloyl chloride (31.4 g) were added. The solution was slowly heated to room temperature (RT) and stirred for 4 h until formation of ethacryloyl chloride droplets. Water was evaporated and residuum was extracted by dichloromethane, the solution was dried with MgSO_4_, filtered through carbon black, and twice recrystallized from acetone/petroleum ether mixture. *N*,*N*′-ethylenebis(acrylamide) was synthetized analogously, only 1,2-diaminoethan (18 g) was used instead of 1-amino-2-propanol.

#### Preparation of Superporouspoly (2-Hydroxyethyl Methacrylate) (HEMA), HEMA-Fn, and Poly [(*N*-(2-Hydroxypropyl) Methacrylamide] (HPMA) Hydrogels

Three polyethylene injection syringes (5 mL) equipped with a stainless filter were filled with needle-like ammonium oxalate crystals (~70 vol %). The monomer mixtures were prepared separately, one for each syringe: (i) HEMA (2.475 g) and EDMA (0.025 g; 1 wt %); (ii) HEMA (2.450 g), EDMA (0.025 g; 1 wt %), and AEMA (0.025 g; 1 wt %); and (iii) HPMA (2.475 g) and EDMAAm (0.025 g; 1 wt %). AIBN (40 mg) was dissolved in 1,4-dioxane (5 mL) and the solution was added to each mixture, which was transferred to the syringe containing crystals. The syringe was closed and the mixture polymerized at 60°C for 16 h. At the end of the reaction, the syringe was cut lengthwise, the hydrogel cylinder was removed and immersed in 10 wt % NH_4_Cl aqueous solution for 24 h, to avoid cracks and to remove 1,4-dioxane. The hydrogel was cut into 2 × 2 × 2 mm cubes, which were washed once with 0.01MHCl (100 mL) for 2 days to remove salts and finally with water. Amino groups of copolymer of HEMA and AEMA were reacted with a solution of GMBS (20 mg) in a mixture of 0.07M PBS (pH 7.4; 10.5 mL) and 1,4-dioxane (5.5 mL) at RT for 30 min to introduce maleimide (MI) groups on the surface. The product was twice washed with PBS/1,4-dioxane solution (PBS/1,4-dioxane = 10.5/5.5 *v*/*v*; 15 mL each), twice with water (15 mL each), and 0.1M PBS (pH 6.8; 15 mL). Fn (1 mg) containing glycine (1.126 mg) and sodium chloride (0.058 mg) were dissolved in PBS buffer (pH 7.4; 2 mL) for 2 h, a solution of TCEP (0.5 mg) in PBS (1 mL) was added and the mixture reacted at 23°C for 40 min. Poly HEMA-MI cubes were incubated in 0.1M PBS (pH 6.8; 20 mL), the solution of reduced Fn was added, and the reaction proceeded at RT for 1 h.

### 4.2. Animal Handling and Surgery

This study was performed in accordance with the European Communities Council Directive of 22 September 2010 (2010/63/EU) regarding the use of animals in research, and was approved by the Ethics Committee of the Institute of Experimental Medicine, Academy of Sciences of the Czech Republic.

### 4.3. Spinal Cord Injury and Hydrogel Implantation

Thirty-four male rats (Wistar, Anlab, Czech Republic) with a weight of 280–400 g, underwent a hemisection at the Th8 level. The animals were intraperitoneally injected with pentobarbital for anesthesia (0.06 g/1 kg i.p.); one dose of ATB (gentamicin 8 mg/1 kg i.m.), atropine (0.08 mg/1 kg s.c.), and mesocain to enhance local anesthesia (1 mg/1 kg s.c. + i.m.) was administered preoperatively. A linear skin incision was performed above the spinous processes of Th7-9; the paravertebral muscles were detached from the laminae Th7-9, and a Th8 laminectomy was performed. The dura was incised longitudinally in the midline and about a 2 mm-segment of spinal cord was dissected in its right half, creating a cavity within the spinal cord tissue. The hydrogel was implanted in such a way as to ensure that it would firmly adhere to the edges of the transection cavity without causing any undue pressure onto the surrounding spinal cord tissue. We implanted 9 rats with HEMA hydrogels with attached fragments of fibronectin, 9 rats with plain HEMA hydrogels, and 9 rats with HPMA hydrogels; 7 animals were left with hemisection only and served as controls. The muscles and skin were sutured again, and the animals were housed two in a cage with food and water ad libitum.

### 4.4. Behavioral Testing

#### BBB Test

The BBB open field test, originally described by Basso, Beattie, and Bresnaham [43] was used to assess basic locomotor functions (joint movement, weight support, forelimb-hindlimb coordination, paw placement, and stability of the body). All 34 rats were used for functional testing throughout the whole evaluation period. The rats were placed on the floor surrounded by boundaries making a rectangular shape once a week. Results were evaluated in the range of 0 to 21 points (0 indicated complete lack of motor capability and 21 movements indicated a healthy rat).

### 4.5. Plantar

The plantar test was performed using the plantar test instrument (Ugo Basile, Italy). A radiant thermal stimulus was applied to the plantar surface of the paws, and the latency of the paw withdrawal response was measured. Each paw was stimulated five times once a week. Hyperalgesia, as a response to the thermal stimulus, was defined as a significant decrease in the withdrawal latency.

### 4.6. Tissue Processing and Histology

The animals were sacrificed 3 months after hydrogel implantation. They were then deeply anesthetized with an intraperitoneal injection of overdose pentobarbital and perfused with physiological saline, followed by 4% paraformaldehyde in 0.1M phosphate buffer. The spinal cord was left in the bone overnight, then removed and postfixed in the same fixative for at least 1 week.

A 4 cm-long segment of the spinal cord with the lesion site in the middle was dissected, and a series of 40 mm-thick longitudinal sections were collected. Hematoxylin–eosin staining was performed, using standard protocols, and the slides were specifically evaluated using an Axio Observer D1 microscope (Carl Zeiss Microimaging GmbH, Oberkochen, Germany). For immunohistochemical studies, the following primary antibodies and dilutions were used: Cy3-conjugated anti-GFAP (1:200; Sigma-Aldrich, Saint Louis, MO, USA) to identify astrocytes, anti-NF 160 (1:200; Sigma-Aldrich, Saint Louis, MO, USA) to identify neurofilaments, and RECA-1 (1:50; Abcam, Cambridge, UK) to identify endothelial cells of blood vessels. Alexa Fluor 594 goat anti–rabbit IgG (1:200; Invitrogen) and Cy3-conjugated anti-mouse IgM (1:100; Invitrogen, Carlsbad, CA, USA) were used as secondary antibodies.

### 4.7. Tissue Quantification

The hydrogels were divided into 3 parts: the cranial end, the central part, and the caudal end. The whole surface of the scaffold per each slice was divided into 6 squares, 2 peripheral cranial and 2 peripheral caudal squares, and the central 2 squares corresponded to the hydrogel center. We calculated the number and the length of axons and blood vessels in each part of the scaffold, using the program TissueQuest Analysis Software (TissueGnostics GmbH, Vienna, Austria). Axonal fibers were manually traced within high resolution mosaic image with second channel as background. Using second channel (488 nm) as a reference channel, the combined image has shifted the background and hydrogel autofluorescence in to brown or yellow shade, whereas the specific fluorescence signal remains clear red. The mosaic was then analyzed by single squares at high magnification using a professional screen. Six spinal cords from each treatment group with 4–5 slices per spinal cord were analyzed. We then combined the data from the peripheral parts of the hydrogels (cranial and caudal ends) and evaluated them together. The central part was quantified and analyzed separately.

### 4.8. qPCR

Expression of rat target genes *Vegfa*, *Bdnf*, *Gap43*, *Gfap*, *Irf5*, *Cd86*, and *Mrc1* at 3 months after hemisection with or without implanted hydrogel (*n* = 3–5), was measured using quantitative real-time reverse transcription polymerase chain reaction (qRT-PCR). RNA was isolated from frozen spinal cord tissue sections, using the High Pure RNA Kit (Roche, Penzberg, Germany). RNA was quantified with spectrophotometer (NanoPhotometerTM P-Class, Munchen, Germany), and isolated RNA was reverse transcribed into cDNA using Transcriptor Universal cDNA Master (Roche, Penzberg, Germany) and a thermal cycler (T100™ Thermal Cycler, Bio-Rad, Hercules, CA, USA). Reactions were performed using cDNA solution, FastStart Universal Probe Master (Roche, Penzberg, Germany), and TaqMan^®^ Gene Expression Assays (Life Technologies, Carlsbad, CA, USA): glyceraldehyde-3-phosphate dehydrogenase/Gapdh/Rn01775763_g1, vascular endothelial growth factor/Vegf/Rn01511602_m1, brain-derived neurotrophic factor/Bdnf/Rn02531967_s1, growth associated protein 43/Gap43/Rn01474579_m1, glial fibrillary acidic protein/Gfap/Rn00566603_m1, interferon regulatory factor 5/Irf5/Rn01500522_m1, macrophage mannose receptor 1/Mrc1/Rn01487342_m1, and CD86/Rn00571654_m1. The final reaction volume was 10 μL containing 25 ng of extracted RNA. Real-time PCR cycler (StepOnePlus™, Life Technologies, Carlsbad, CA, USA) was used for amplification. The following cycling conditions were used, 2 min at 50 °C, 10 min at 95 °C, followed by 40 cycles of 15 s at 95 °C and 1 min at 60 °C. Relative quantification of gene expression was determined using the ΔΔCt method. Data was analyzed with StepOnePlus^®^ software (v2.3) Life Technologies, Carlsbad, CA, USA). For normalization of gene expression levels Gapdh gene was used. A log2 scale was used to display the symmetric magnitude for up- and downregulated genes. From obtained values of control animals (hemi-section only), the arithmetical mean was calculated and this value was set as zero. The statistical analysis (*t*-test) was performed from ΔCt values of controls as well as treated animals.

### 4.9. Statistical Analysis

The mean values are reported as mean ± SEM. For behavioral tests histological analysis and gene markers intergroup differences were analyzed using one-way ANOVA (probability values <0.05).

## 5. Conclusions

The HPMA hydrogel shows better potential for SCI repair compared to HEMA hydrogel. The attachment of fibronectin improves limited connective tissue and axonal ingrowth, however without any long-term functional improvement. More sophisticated modifications of future scaffolds would be needed if we want to achieve functionally relevant long-term results.
